# Inflammatory cytokine and acute phase protein concentrations in the peripheral blood and uterine washings of cows with subclinical endometritis in the late postpartum period

**DOI:** 10.1007/s11259-015-9635-4

**Published:** 2015-04-08

**Authors:** Piotr Brodzki, Krzysztof Kostro, Leszek Krakowski, Jan Marczuk

**Affiliations:** Department and Clinic of Reproduction, University of Life Sciences in Lublin, ul. Głęboka 30, 20-612 Lublin, Poland; Department of Epizootiology and Clinic of Infections Diseases, Faculty of Veterinary Medicine, University of Life Sciences in Lublin, Głęboka 30, 20-033 Lublin, Poland; Department and Clinic of Internal Medicine, University of Life Sciences in Lublin, ul. Głęboka 30, 20-612 Lublin, Poland

**Keywords:** Acute phase proteins, Cows, Cytokine, Subclinical endometritis

## Abstract

The aim of the study was to evaluate the concentrations of proinflammatory cytokines: tumor necrosis factor (TNF-α) and interleukin-6 (IL-6), anti-inflammatory cytokine interleukin-10 (IL-10), and acute phase proteins (APPs)—haptoglobin (Hp) and serum amyloid A (SAA) in serum and uterine washings of cows with subclinical endometritis, and compare them to healthy animals. The study was performed on 24 cows on day 60 after delivery. The cows were divided into two groups based on the results of cytological tests: 12 cows with subclinical endometritis and 12 healthy cows. Experimental material consisted of blood serum and uterine washings. The levels of the following cytokines in the study material were determined with ELISA: TNF-α, IL-6, IL-10 and APPs - Hp and SAA. The results show that the levels of TNF-α (*p* < 0.01), IL-6, IL-10 as well as SAA and Hp were significantly higher in the serum of cows with subclinical endometritis compared to the controls (*p* < 0.001). Uterine washings had significantly higher levels of IL-6, IL-10, and Hp in the experimental cows compared to the controls (*p* < 0.001). The demonstrated differences in the concentration of cytokines and APP between cows with subclinical endometritis and healthy cows, in both the serum and uterine washings, may suggest the usefulness of these parameters in the diagnosis of subclinical endometritis in cows in the late postpartum period.

## Introduction

Subclinical endometritis compared to other types of inflammation of the uterus, is diagnosed late due to the lack of noticeable clinical symptoms related to the reproductive system, usually when insemination becomes ineffective. Despite numerous studies, the implementation of new diagnostic methods and the use of different therapeutic methods, endometritis in dairy cows remains a serious economic problem all over the world. This is mainly due to large economic losses caused by the low rate of artificial insemination and the necessity to cull animals in the herd (Galvao 2012; LeBlanc 2008 and Lee and Kim 2007). Bacterial infections play an important role in the complex etiology of subclinical endometritis (Bicalho et al. 2010; Földi et al. 2006 and Gautam et al. 2010). Although more than 70 % of cows clear uterine bacteria via innate immune responses, 17 to 37 % of cows develop clinical endometritis, whereas 14 to 53 % develop subclinical endometritis (Cheong et al. 2011; Gilbert et al. 2005; Kim and Kang 2003 and Madoz et al. 2014)

Cellular and humoural mechanisms of local non-specific and specific immunity play an essential role in the elimination of uterine infection. The development of bovine endometritis is associated with very complex signaling processes involving the detection of bacterial components by innate immune cells *via* Toll-like receptors, the production of the tumor necrosis factor-α (TNF-α) and other proinflammatory cytokines (e.g., interleukins (IL)), and the mobilization of neutrophils followed by the phagocytosis of invading pathogens within the uterine lumen (Beutler et al. 2003; Herath et al. 2009; Sheldon et al. 2009 and Turner et al. 2012). Proinflammatory cytokines (e.g., TNF-α, IL-1β, and IL-6) and chemokines (e.g., IL-8) stimulate neutrophil and monocyte diapedesis and chemoattraction and promote increased phagocytosis (Butterfield et al. 2006 and Singh et al. 2008).

Pro-inflammatory cytokines are also potent stimulators for the production of acute phase proteins (APPs), such as haptoglobin (Hp), acid glycoprotein, ceruloplasmin or serum amyloid A (SAA) (Tothova et al. 2008). Their task is to assist in the elimination of infection, e.g., through the modulation of other immune proteins or stimulation of phagocytosis. The protective function of APPs against the damaging effects of enzymes formed during the inflammatory response that can lead to organ damage, is also of significant importance. Acute phase proteins are produced in the liver, and their concentration in the blood serum of cows increases over the first few weeks after birth, in response to uterine infection caused by microorganisms (Sheldon et al. 2001 and Tothova et al. 2008). Although there is a possibility that the synthesis of APPs takes place outside the liver, their presence in the uterine endometrium cells of cows was not confirmed in vitro (Davies et al. 2008). However, research conducted by Chapwanya et al. (2013) has suggested that the production of serum amyloid A by endometrium cells in cows is possible.

Research conducted by Galvao et al. (2011) and Gashemi et al. (2012) showed that the expression of mRNA of inflammatory cytokines in uterine tissue was related to the development of bovine clinical or subclinical endometritis. Other studies have also demonstrated significant differences in the serum levels of pro-inflammatory cytokines between cows with endometritis and healthy individuals (Islam et al. 2013 and Kasimanickam et al. 2013). A higher expression of the genes of proinflammatory cytokines in endometrial tissue and/or an increase in the levels of these cytokines in the serum is considered to be a sensitive prognostic indicator of the development of endometritis in cows (Galvão et al. 2011; Ghasemi et al. 2012; Islam et al. 2013 and Kasimanickam et al. 2013). Recent studies have shown that the assessment of the level of proinflammatory cytokines is possible not only in uterine tissue and serum, but also in the liquid collected from the uterus. Cytokine levels in uterine washings have been shown to be significantly higher in cows with clinical endometritis than in healthy animals (Kim et al. 2014). The listed uterine washing tests have shown only slight changes in the concentration of TNF-α, IL-6 and IL-10 in cows with subclinical endometritis. These are the only studies of this kind, covering the period from 4 to 8 weeks after birth. We still do not know whether these cytokines may be a diagnostic tool for subclinical endometritis in dairy cows. In addition, it is still not known whether it is possible to assess the risk of inflammation of the uterus on the basis of acute phase proteins in both the serum and uterus. At present, it is also unknown whether APPs can be produced in the uterus.

The objective of the study was to evaluate the concentrations of the following proinflammatory cytokines: TNF-α, IL-6, anti-inflammatory cytokine IL-10, and acute phase proteins – Hp and SAA in the serum and uterine washings of cows with subclinical endometritis and healthy animals in the late postpartum period (60 DPP).

## Materials and methods

### Animals

The study was approved by the Local Ethics Committee at the University of Life Sciences in Lublin. The examinations were performed on 24 dairy Holstein-Friesian cows between the second and third lactation. The cows were choosen from one herd in which feeding was based on the TMR (Total Mixed Ration) system and included maize silage, grass silage, hay, straw, grain meal, soy meal, mineral supplements and protein supplements. The nutrition of cows was adjusted to actual milk productivity and the gestation period. Reproductive system control was conducted regularly at monthly intervals by rectal examination combined with ultrasonography. Cows with no complications during parturition and no signs of inflammation were applied the synchronization protocol of estrus and ovulation (presynch-ovsynch protocol) and artificial insemination (AI) with frozen semen. The cows with uterine inflammation were properly treated, and subsequently subjected to the synchronization protocol of estrus and ovulation and AI. The cows with ovarian cycle disturbances were treated individually according to the cause diagnosed. Investigations were conducted from February 2012 to March 2013.

### Experimental design

Initially, 36 cows with clinical signs of endometritis were included. These animals were treated with intrauterine infusion of cefaphirin (Metricure®, Intervet International B. V, Boxmeer, Netherlands) between 21-47 DPP. On day 60 after parturition, 8 out of 36 cows showed clinical signs of endometritis; cytological examination revealed subclinical endometritis in 12 cows, which were studied further. From the group of 16 healthy cows without any signs of clinical or subclinical endometritis, 12 animals were selected as the control group. Their Body Condition Score was 3.5 ± 0.5, and they produced 34.2 ± 5.4 litres of milk per day during the last lactation period. The study included the assessment of the clinical health status of animals and a detailed evaluation of the reproductive system by means of ultrasound using a Honda 1500 Ultrasound (Honda Electronics CO., LTD., Toyohashi, Japan) with a dual frequency 5.0/7.5 MHz intrarectal transducer. In addition, cytological tests were performed of smears from the uterus. The tests were conducted on day 60 after delivery. Twelve cows with subclinical endometritis were selected based on the uterine cytology (neutrophil count >5 %) – they represented the experimental group. The control group consisted of 12 cows without inflammation of the uterus (neutrophil count <5 %). The selection process of cow was based on literature (Gilbert et al. 2005 and Sheldon et al. 2006). Cytological and immunological tests were also performed in both groups of animals. The biological material collected for laboratory tests was sent to the laboratory within an hour after sampling.

### Cytological tests

The material for cytological tests was collected with an intrauterine brush adapted for cows (Directa pro, Jiangsu Yada Technology Group Co., Ltd, Jiangsu, China). Imprint cytology specimens were prepared from the cytological brushes, which were fixed and stained by means of the Hemacolor method after drying (Merck, Darmstadt, Germany). The preparations were evaluated under a microscope (CX 41, Olympus Corporation, Tokyo, Japan) at 1000x magnification; the number of neutrophils was counted according to protocols available in the literature (Kasimanickam et al. 2004, 2005 and Sheldon et al. 2006).

### Sampling of blood and uterine washings

The material for immunological analysis included peripheral blood and uterine washings. Blood samples (9 ml) were collected from the external jugular vein into clot activator tubes, whereas uterine washings were collected into Vacutest standard tubes (Vacutest Kima srl, Arzergrande (PD), Italy). Blood samples were then centrifuged at 2500 x g for 10 min at 4 °C, and the serum was harvested and transferred to 2-ml microcentrifuge tubes and stored at -80 °C until analysis.

Fifty ml of isotonic phosphate-buffered saline (PBS) was injected into the uterus and washings were collected using a Foley catheter (Jorgen Kruuse, Marslev, Denmark) with a syringe. The uterus was massaged through the rectum for a few minutes, and the saline solution was aspirated by pulling back the syringe plunger to create negative pressure in the catheter. In most cases, approximately 40 ml of fluid was recovered, while some of it remained in the uterus. The uterine fluid was centrifuged at 2500 x g for 10 min at 4 °C, and the supernatant was transferred to 2-ml microcentrifuge tubes and stored at -80 °C until analysis.

### Measurements of cytokines in uterine washings and blood serum

The concentrations of TNF-α, IL-6 and IL-10 in blood serum and uterine flush samples were determined using commercially available kits, i.e., bovine enzyme-linked imunosorbent assay (ELISA) kits for TNF-α, IL-6 and IL-10 (USCN Life Science Inc., Houston, USA). The inter- and intra-assay coefficients of variation (CV) for all examined cytokines were <12 and <10 %, respectively. All procedures were performed according to the guidelines provided by the manufacturers and methods in the literature (Kim et al. 2014). Absorbance readings were performed on an automatic microplate reader (Asys Expert Plus, Biochrom Ltd., Cambridge, England) at 450 nm.

### Measurements of acute phase proteins in uterine washings and blood serum

Measurements of the level of serum amyloid A in blood serum and uterine flush was performed using a commercial ELISA kit (Tridelta Development Ltd., Maynooth, Kildare, Ireland). The inter- and intra-assay coefficients of variation for SAA analysis were <12.1 and <7.5 %, respectively. The determination of haptoglobin in blood serum and uterine flush was performed using a commercial colorimetric assay kit (Tridelta Development Ltd., Kildare, Ireland). The inter- and intra-assay CVs for Hp analysis were <5.7 and <6.3 %, respectively. Procedures were performed according to the manufacturers’ instructions and literature methods (Suojala et al. 2008 and Tothova et al. 2012). Absorbance readings and subsequent calculations of final concentrations were performed on an automatic microplate reader (Asys Expert Plus, Biochrom Ltd., Cambridge, England) at 450 nm, 630 nm for Hp, and 630 nm as a reference for SAA. Lyophilized bovine acute phase serum was used as a standard; calibration was performed according to the European Union concerted action on standardization of animal APPs (No. QLK5-CT-1999-0153).

### Statistical analysis

All values are presented as means ± SEM. Statistical analysis was performed using the Statistica software (version 10.0). Data was found to be normally distributed, as demonstrated by the Kolomogorov-Smirnov test and Lillefors correction. Mean values were compared between healthy and subclinical endometritis cows and between blood serum and uterine washings in the two groups, using Student *t*-test. P-value ≤0.01 was considered as statistically significant.

## Results

**Fig. 1:**
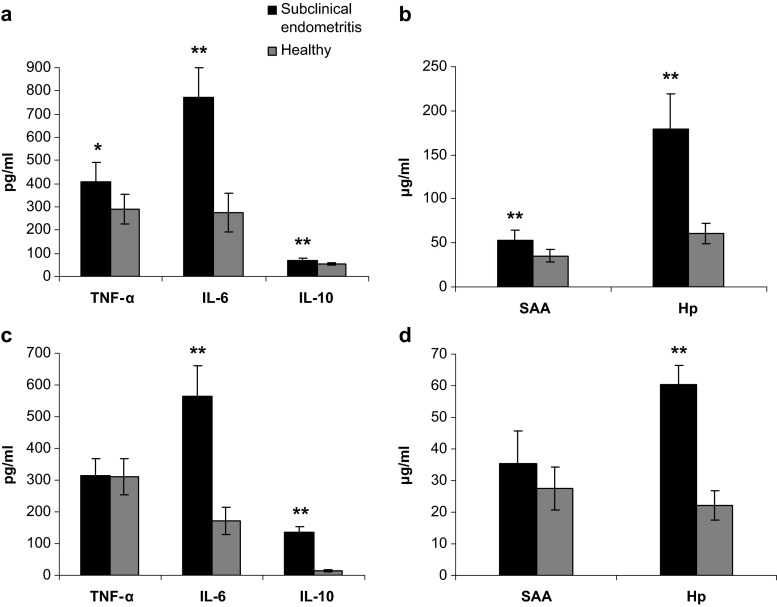
Levels of tumor necrosis factor (TNF-α), interleukin 6 (IL-6), interleukin 10 (IL-10), and haptoglobin (Hp), serum amyloid A (SAA) in blood serum (**a**, **b**) and uterine washings (**c**, **d**) of cows with subclinical endometritis (*n* = 12) and healthy cows (*n* = 12). Mean ± SEM, statistical significance at * - *p* ≤ 0.01 and ** - *p* ≤ 0.001, compared with the control

The data shows that in the cows with subclinical endometritis the mean leukocyte count was 12.46 ± 3.22 %; this was significantly higher (*p* < 0.001) than for healthy cows (3.14 ± 1.24 %). The results of the levels of pro-inflammatory cytokines - TNF-α and IL-6, anti-inflammatory IL-10, and acute phase proteins - SAA and Hp in the blood serum and uterine washings of cows with subclinical endometritis and healthy individuals are presented in Fig. 1. The data shows that the levels of TNF-α (*p* < 0.01), IL-6, IL-10 (68.73 ± 12.15 pg/ml), as well as acute phase proteins (SAA and Hp) in the serum of cows with subclinical endometritis were significantly higher compared to healthy cows (IL-10 53.59 ± 3.19 pg/ml) (*p* < 0.001) (Fig. 1a and b). The uterine washings in cows with subclinical endometritis had significantly higher levels of IL-6, IL-10, and Hp compared to the healthy group (*p* < 0.001) (Fig. 1c and d). The serum levels of TNF-α and IL-6 (*p* < 0.01) as well as Hp and SAA (*p* < 0.001) were higher in cows with subclinical endometritis, while the levels of IL-10 were lower (*p* < 0.001) compared to the uterine washings. In the control cows, IL-6, IL-10, Hp (*p* < 0.001) and SAA (*p* < 0.01) were higher in the serum than in uterine washings.

## Discussion

The aim of the study was to evaluate the concentrations of proinflammatory cytokines: TNF-α and IL-6, anti-inflammatory cytokine IL-10, and APPs - Hp and SAA, in the serum and uterine washings of cows with subclinical endometritis and healthy animals. Studies in both groups of cows were carried out on day 60 after birth (DPP). This is the first study of this type conducted on uterine washings of cows in the late postpartum period. The higher levels of inflammatory cytokines TNF-α, IL-6 and IL-10, and acute phase proteins, Hp and SAA presented in our study in the serum of cows in the experimental group may be associated with subclinical inflammation of the uterus. Increased activity of immunocompetent cells, stimulated mainly in the uterus, but also in peripheral blood, could be the cause of an increase in the concentration of inflammatory mediators in the blood. The majority of microorganisms are eliminated from the uterus in the early postpartum period to 21 DPP, while the uterus should be already sterile at 6–8 weeks after birth (Sheldon et al. 2009). When the number of pathogens is low or they are absent, local immune mechanisms are not activated or are active at a low level. The concentration of inflammatory mediators (cytokines, chemokines, APPs) also does not increase, which was observed in healthy cows. In contrast, cows with endometritis with persistent inflammation of the uterus, showed an increase in these mediators. Research conducted by Kasimanickam et al. (2013) are consistent with our results and confirm the higher levels of proinflammatory cytokines (TNF-α, IL-1β and IL-6) in blood serum of cows with endometritis compared to healthy cows. However, the study by Kim et al. (2014) showed no difference in the concentration of these cytokines in the serum of cows with endometritis and healthy animals. Ishikawa et al. (2004) also did not observe significant changes in the level of IL-6 in the serum of cows with endometritis.

We have shown in our study significantly higher levels of IL-6, IL-10, and Hp in uterine washings of cows with endometritis compared to cows without the disease. In addition, we have also demonstrated that cows with endometritis had a 2-fold higher concentration of IL-10 in uterine washings than in blood serum. One of the reasons for the high concentration of IL-6 was the secretion of this protein by uterine endometrium cells, as a result of recognition of the pathogen associated molecular patterns (PAMPs) by specific Toll-like receptors (TLR) (Beutler et al. 2003; Sheldon et al. 2009 and Turner et al. 2012). This reaction is a key activation element of the local immune response of the uterus, leading to leucocyte chemotaxis and enhancement of the phagocytic activity of neutrophils and macrophages already present in the uterus. Leukocytes present in the uterus and those flowing in with blood, which were activated in this manner or directly stimulated by PAMPs of microorganisms (mainly phagocytes), also secrete cytokines, contributing to an increase in their concentration in the uterus (Singh et al. 2008). Our previous studies (Brodzki et al. 2014a, 2014b) and works of many other authors (Kasimanickam et al. 2004, 2005 and Sheldon et al. 2006) confirmed the presence of pathogens and the consequent higher number of leukocytes (mainly neutrophils) in the uterus of cows with subclinical endometritis. This is reflected in the results of our research in the form of elevated levels of proinflammatory IL-6 in uterine washings of cows with endometritis. Other studies also showed a higher level of IL-6 mRNA in the uterus of cows with subclinical endometritis compared to healthy cows (Loyi et al. 2013). In contrast, studies by Kim et al. (2014) demonstrated a lack of increase in IL-6 production in cows with subclinical endometritis, but only in cows with clinical endometritis. The increase of anti-inflammatory IL-10, presumably results from the increasing level of T CD8+ lymphocytes during the persistent inflammation of the uterus that may have suppressor functions by inhibiting inflammatory reactions. Our previous studies confirmed the percentage increase of CD8+ cells in uterine washings of cows with subclinical endometritis (Brodzki et al. 2014a, 2014b). CD8+ lymphocytes, by secreting IL-10, play a protective role in endometrial tissue by suppressing the immune-effector cells, which attenuates autoimmunological response (Banos et al. 2013; Park et al. 2004 and Shafer-Weaver and Sordillo 1997). Our studies also showed that such interaction may be associated with the weakening of the local anti-infectious immunity and the lack of complete elimination of microorganisms from the uterus. The result is the long-term persistence of endometritis.

Acute phase proteins (APPs) play an important role in different stages of the inflammatory response, and thus may serve as markers of various types of diseases in cattle (Maden et al. 2012 and Tothova et al. 2012). Of the various APPs occurring in cows, haptoglobin (Hp) and serum amyloid A (SAA) are the primary positive biomarkers (Tothova et al. 2014). SAA is an apolipoprotein occurring 24–48 h after infection, as a protein of the first line of response, and its secretion is dependent on IL-1 and/or TNF-α (Petersen et al. 2004 and Tothova et al. 2014). Hp in turn is a protein of the second line of response, whose secretion is regulated by IL-6, and its high level is characteristic to long and less severe inflammatory processes (Petersen et al. 2004 and Tothova et al. 2014). Our findings seem to confirm these relationships, as the level of SAA was significantly higher in the serum of cows with endometritis compared to healthy animals. At the same time, a high level of TNF-α was detected in cows with subclinical endometritis. The level of haptoglobin (Hp) was significantly higher both in serum and uterine washings in cows with endometritis. Such changes in the concentration of APPs can suggest persistence of chronic inflammation of the uterus. The persistence of high concentrations of Hp in the serum and uterine washings of cows with endometritis is very interesting from a clinical point of view. This protein can be considered a marker of inflammation of the uterus in this species. The increase of Hp in uterine washings is difficult to explain, because the local production of this protein in endometrium cells has not been confirmed. However, a significant increase of its level may suggest local production of Hp in the course of endometritis. Another possibility is the penetration of the protein into the lumen of the uterus from the blood, where significantly higher levels of this protein were recorded compared to uterine washings.

Our results indicate that the evaluation of the levels of cytokines and Hp in serum and uterine washings can be an important diagnostic indicator of endometritis in cows. Furthermore, with an increase of anti-inflammatory cytokines, the possible persistence or further development of inflammation of the uterus may be predicted. However, the condition for the correct assessment of the direction of the development of inflammation and the closely related mechanisms of local immunity of the uterus is a simultaneous examination of the concentration of several related cytokines with antagonistic interaction. This way, it is possible to accurately determine the current state of the local resistance of the uterus, which largely facilitates the determination of further prophylactic or therapeutic treatment.

## Conclusion

The presented study shows that the level of each cytokine and APP in serum was higher in cows with subclinical endometritis compared to healthy cows. However, uterine washings demonstrated higher levels of IL-6, IL-10 and HP in cows with subclinical endometritis than healthy cows. Based on these results, we can assume that the assessment of the levels of these cytokines and acute phase proteins can be an important diagnostic indicator of subclinical endometritis in dairy cows. Simultaneously the evaluation of selected indicators of the antagonistic interaction can be helpful in determining the current state of local immunity of the uterus, and facilitate the determination of further prophylactic or therapeutic treatment.

## References

[CR1] Banos G, Wall E, Coffey MP, Bagnall A, Gillespie S, Russel GC, McNelly TN (2013). Identification of immune traits correlated with dairy cow health, reproduction and productivity. PLoS One.

[CR2] Beutler B, Hoebe K, Du X, Ulevith RJ (2003). How we detect microbes and respond to them: toll-like receptors and their transducers. J Leukoc Biol.

[CR3] Bicalho RC, Machado VS, Bicalho ML, Gilbert RO, Teixeria AG, Caixeta LS, Pereira RV (2010). Molecular and epidemiological characterization of bovine intrauterine Escherichia Coli. J Dairy Sci.

[CR4] Brodzki P, Kostro K, Brodzki A, Lisiecka U (2014). Determination of selected parameters for non-specific and specific immunity in cows with subclinical endometritis. Anim Reprod Sci.

[CR5] Brodzki P, Kostro K, Brodzki A, Lisiecka U, Kurek Ł, Marczuk J (2014). Phenotyping of leukocytes and granulocyte and monocyte phagocytic activity in the peripheral blood and uterus of cows with endometritis. Theriogenology.

[CR6] Butterfield TA, Best TM, Merrick MA (2006). The dual roles of neutrophils and macrophages in inflammation: a critical balance between tissue damage and repair. J Athl Train.

[CR7] Chapwanya A, Meade KG, Doherty ML, Callanan JJ, O’Farrelly C (2013). Endometrial epithelial cells are potent producers of tracheal antimicrobial peptide and serum amyloid A3 gene expression in response to E. coli stimulation. Vet Immunol Immunopath.

[CR8] Cheong SH, Nydam DV, Galvão KN, Crosier BM, Gilbert RO (2011). Cow-level and herd-level risk factors for subclinical endometritis in lactating Holstein cows. J Dairy Sci.

[CR9] Davies D, Meade KG, Herath S, Eckersall PD, Gonzalez D, White JO, Conlan RS, O’Farrelly C, Sheldon IM (2008). Toll-like receptor and antimicrobial peptide expression in the bovine endometrium. Reprod Biol Endocrinol.

[CR10] Földi J, Kulcsár M, Pécsia A, Huyghe B, de Sa C, Lohuis JA, Cox P, Huszenicza G (2006). Bacterial complications of postpartum uterine involution in catle. Anim Reprod Sci.

[CR11] Galvão KN (2012). Postpartum uterine diseases in dairy cows. Anim Reprod.

[CR12] Galvão KN, Santos NR, Galvão JS, Gilbert RO (2011). Association between endometritis and endometrial cytokine expression in postpartum Holstein cows. Theriogenology.

[CR13] Gautam G, Nako T, Koike K, Long ST, Yusuf M, Ranasinghe RM, Hayashi A (2010). Spontaneous recovery or persistence of postpartum endometritis and risk factors for its persistence in Holstein cows. Theriogenology.

[CR14] Ghasemi F, Gonzalez-Cano P, Griebel PJ, Palmer C (2012). Proinflammatory cytokine gene expression in endometrial cytobrush samples harvested from cows with and without subclinical endometritis. Theriogenology.

[CR15] Gilbert RO, Shin ST, Guard CL, Erb HN, Frajblat M (2005). Prevalence of endometritis and its effects on reproductive performance of dairy cows. Theriogenology.

[CR16] Herath S, Lilly ST, Fischer DP, Williams ER, Dobson H, Bryant CE, Sheldon IM (2009). Bacterial lipopolysaccharide induces an endocrine switch from prostaglandin F2alpha to prostaglandin E2 in bovine endometrium. Endocrinology.

[CR17] Ishikawa Y, Nakada K, Hagiwara K, Kirisawa R, Iwai H, Moriyoshi M (2004). Changes in interleukin-6 concentration in peripheral blood of pre- and post-partum dairy cattle and its relationship to postpartum reproductive diseases. J Vet Med Sci.

[CR18] Islam R, Kumar H, Nandi S, Rai RB (2013). Determination of anti-inflammatory cytokine in periparturient cows for prediction of postpartum reproductive diseases. Theriogenology.

[CR19] Kasimanickam R, Duffield TF, Foster RA, Gartley CJ, Leslie KE, Walton JS, Johnson WH (2004). Endometrial cytology and ultrasonografy for the detection of subclinical endometritis in postpartum dairy cows. Theriogenology.

[CR20] Kasimanickam R, Duffield TF, Foster RA, Gartley CJ, Leslie KE, Walton JS, Johnson WH (2005). A comparison of the cytobrush and uterine lavage techniques to evaluate endometrial cytology in clinically normal postpartum dairy cows. C Vet J.

[CR21] Kasimanickam RK, Kasimanickam VR, Olsen JR, Jeffress EJ, Moore DA, Kastelic JP (2013). Associations among serum pro- and anti-inflammatory cytokines, metabolic mediators, body condition, and uterine disease in postpartum dairy cows. Reprod Biol Endocrinol.

[CR22] Kim IH, Kang HG (2003). Risk factors for postpartum endometritis and the effect of endometritis on reproductive performance in dairy cows in Korea. J Reprod Dev.

[CR23] Kim IH, Kang HG, Jeong JK, Hur TY, Jung YH (2014). Inflammatory cytokine concentrations in uterine flush and serum samples from dairy cows with clinical or subclinical endometritis. Theriogenology.

[CR24] LeBlanc SJ (2008). Postpartum uterine disease and dairy herd reproductive performance: a reviw. Vet J.

[CR25] Lee JI, Kim IH (2007). Pregnancy loss in dairy cows: the contributing factors, the effects on reproductive performance and the economic impact. J Vet Sci.

[CR26] Loyi T, Kumar H, Nandi S, Mathapati BS, Patra MK, Pattnaik B (2013). Different expression of pro-inflammatory cytokines in endometrial tissue of buffalos with clinical and sub-clinical endometritis. Res Vet Sci.

[CR27] Maden M, Ozturk AS, Bulbul A, Avci A, Yazar E (2012). Acute-phase proteins, oxidative stress, and enzyme activities of blood serum and peritoneal fluid in cattle with abomasal displacement. J Vet Intern Med.

[CR28] Madoz LV, Giuliodori MJ, Migliorisi AL, Jaureguiberry M, de la Sota RL (2014). Endometrial cytology, biopsy, and bacteriology for the diagnosis of subclinical endometritis in grazing dairy cows. J Dairy Sci.

[CR29] Park YH, Joo YS, Park JY, Moon JS, Kim SH, Kwon NH, Ahn JS, Davis WC, Davies CJ (2004). Characterization of lymphocyte subpopulations and major histocompatibility complex haplotypes of mastitis-resistant and susceptible cows. J Vet Sci.

[CR30] Petersen HH, Nielsen JP, Heegaard PMH (2004). Application of acute phase protein measurements in veterinary clinical chemistry. Vet Res.

[CR31] Shafer-Weaver KA, Sordillo LM (1997). Bovine CD8+ suppressor lymphocytes alter immune responsiveness during the post-partum period. Vet Immunol Immunopath.

[CR32] Sheldon IM, Noakes DE, Rycroft A, Dobson H (2001). Acute phase protein response to postpartum uterine bacterial contamination in cattle. Vet Rec.

[CR33] Sheldon IM, Levis GS, LeBlanc S, Gilbert RO (2006). Defining postpartum uterine disease in cattle. Theriogenology.

[CR34] Sheldon IM, Cronin J, Goetze L, Donofrio G, Schuberth HJ (2009). Defining postpartum uterine disease and the mechanisms of infection and immunity in the female reproductive tract in cattle. Biol Reprod.

[CR35] Singh J, Murray RD, Mshelia G, Woldehiwet Z (2008). The immune status of the bovine uterus during the peripartum period. Vet J.

[CR36] Suojala L, Orro T, Järvinen H, Saatsi J, Pyörälä S (2008). Acute phase response in two consecutive experimentally induced E.coli intramammary infections in dairy cows. Acta Vet Scand.

[CR37] Tothova CS, Nagy O, Seidel H, Konvicna J, Farkasova Z, Kovac G (2008). Acute phase proteins and variables of protein metabolism in dairy cows during the pre- and postpartial period. Acta Vet Brno.

[CR38] Tothova CS, Nagy O, Seidel H, Kovac G (2012). Acute phase proteins in relation to various inflammatory diseases of calves. Comp Clin Pathol.

[CR39] Tothova CS, Nagy O, Seidel H, Kovac G (2014). Acute phase proteins and their use in the diagnosis of diseases in ruminants: a reviw. Vet Med-Czech.

[CR40] Turner ML, Healey GD, Sheldon IM (2012). Immunity and inflammation in the uterus. Reprod Dom Anim.

